# Studies on the Urinary Excretion of Certain Tryptophan Metabolites before and after Tryptophan Loading Dose in Bilharziasis, Bilharzial Bladder Cancer and Certain Other Types of Malignancies in Egypt

**DOI:** 10.1038/bjc.1964.68

**Published:** 1964-09

**Authors:** A. S. Khalafallah, M. A. M. Abul-Fadl


					
592

STUDIES ON THE URINARY EXCRETION OF CERTAIN TRYPTO-

PHAN AIETABOLITES BEFORE AND AFTER TRYPTOPHAN
LOADING DOSE IN BILHARZIASIS, BILHARZIAL BLADDER
CANCER AND CERTAIN OTHER TYPES OF MALIGNANCIES
IN EGYPT

A. S. KHALAFALLAH AND M. A. M. ABUL-FADL

From the Department of Chemical Pathology, Faculty of Medicine,

Cairo University, UAR

Received for publication March 7, 1964

THE significance of certain tryptophan metabolites and their possible role in
the production of bladder cancer was first suggested by Dunning, Curtis and Maun
(1950), while studying the carcinogenic effect of 2-acetylaminofluorine on rat
bladder. Bladder implantation experiments conducted by Allen, Boyland, Dukes,
Horning and Watson (1957) have proved the carcinogenic nature of these trypto-
phan metabolites. Such metabolites were also reported to be present in abnor-
mally high concentration in patients suffering from various malignancies particu-
larly the bladder cancers (Dalgliesh, 1955; Boyland and Watson, 1956; Price,
1958). The abnormal excretion was made clearer when a single dose of L-trypto-
phan was given to such patients.

The present work is essentially an extension of the preliminary study previously
reported in this Journal (Abul-Fadl and Khalafallah, 1961) concerning the urinary
excretion of such tryptophan metabolites by bilharzial and bladder cancer patients
in Egypt. The tryptophan loading test has been found useful, and the study was
extended to include other cases of malignancy occurring in this country. It
would be interesting to find out by such an investigation any possible climatic,
nutritional or racial variations in the excretion of these metabolites that might
help to explain the abnormally high incidence of certain malignant diseases among
the Egyptians, particularly bladder cancer. The latter disease is not so common
in other countries which are known to be similarly affected by bilharziasis.

The tryptophan metabolites (anthranilic acid, 3-hydroxy-anthranilic acid,
kynurenine, kynurenic acid, xanthurenic acid, indole-3-acetic acid and 5-hyroxy-
indole-acetic acid); were determined in urines collected before and after the oral
administration of a single dose of 100 mg. of L-typrophan/kg. body weight.

The results obtained showed characteristic patterns of excretion of these
tryptophan metabolites in urines collected from bilharzial patients, bilharzial
bladder cancers, and patients suffering from twelve other different types of
malignant disease of relatively common occurrence in Egypt.

The tryptophan loading test has proved its value in assessing such urinary
excretion patterns.

EXPERIMENTAL

Choice of cases

All cases included in this investigation were carefully selected and examined in
the medical, surgical, urology and X-ray departments of the Cairo University

URINARY EXCRETION OF TRYPTOPHAN METABOLITES

Hospitals (Kasr El-Aini). The diagnosis was reached after careful clinical,
X-ray and laboratory investigationis. The normal subjects (100 cases) were aged
from 18 to 55 years and were mostly without known previous history of bilharzial
infection. Urines and stools were examined and proved free from any other
parasitic infections.

The bilharzial cases comprised simple and complicated infections. The simple
(100 cases), represent the first attack of urinary schistosomiasis. The complicated
bilharziasis (100 cases) represent victims of repeated attacks of schistosomiasis
accompanied by hepatosplenomegaly.

The bladder cancer cases (100 cases) had a definite bilharzial history. Crysto-
scopic examinations and laboratory tests on biopsies revealed the presence of
bilharzial granulomatous tissues.

Twelve types of malignancy involving tissues other than the bladder, breast
(8 cases), cervix (5 cases), prostate (5 cases), lip (5 cases), larynx (4 cases), thyroid
(4 cases), tongue (4 cases), brain (3 cases), bone (5 cases), liver (5 cases), bronchuis
(5 cases), and rectum (4 cases).
Collection of urine

Complete 24 hour urines collected under toluene and preserved in the cold
whenever possible, were subjected to routine examination and chromatographic
studies. For the determination of 5-hydroxyindole acetic acid, the urines were
collected over a mixture of glacial acetic acid and toluene (25 ml. + 5 ml. respec-
tively). Cold storage was also advisable.

In the tryptophan loading test, the fasting patients were given a single dose of
100 mg. L-tryptophan per kg. body weight suspended in gum acacia syrup. The
urine was then collected during the following 24 hour period.

METHODS

Colorimetric Determinations

5-Hydroxyindole acetic acid was determined according to the method described
by MacFarlane, Dalgliesh, Dutton, Lennox, Nyhus and Smith (1956). For indole-
3-acetic acid, the method of Weissbach, King, Sjoerasmo and Uden Friend (1959)
was adopted.

The diazotisable aromatic amines (kynurenine and anthranilic acid) and the
orthohydroxyamine (3-hydroxyanthranilic acid) were determined by the technique
derived by Tompsett (1959). For the determination of kynurenic acid and
xanthurenic acid, the method of Satoh and Price (1958) was used.

Chrornatographic Study

The technique described by Dalgliesh (1955) and modified by Boyland and
Williams (1956) was applied as follows:

1/50th of the complete 24 hour urine collection was acidified with acetic acid
to about pH 4, then centrifuged. The clear supernatant was transferred in 10 ml.
portions to a glass tube column 1 cm. in internal diameter, 15 cm. in length,
previously prepared to contain a layer of deactivated charcoal one cm. long
suspended in 0.500 acetic acid solution over a plug of glass wool. After passing
the whole amount of urine through the charcoal, the column was washed with 50 ml.

593

A. S. KHALAFALLAH AND M. A. M. ABUL-FADL

distilled water, passing aliquots of 10 ml. at a time. Elution of the adsorbed
metabolites was then effected by 50 ml. 5% (w/v) aqueous phenol solution also
in 10 ml. aliquots.

The eluate was evaporated to dryness under reduced pressure, 10 ml. of pure
methanol were added to the residue, stirred, then evaporated again to dryness.
The residue was finally dissolved in one ml. distilled water. Using the butanol-
acetic acid water system (4:1: 5) (Partridge 1948), the paper chromatographic
study was then conducted on 0*05 ml. of this solution. Two standard mixtures,
containing 2 and 10 micrograms respectively of each of the investigated metabolites
in a total volume of 0-05 ml. were also run parallel to the tested urines on the same
chromatograms. After drying at 40? c., the spots of kynurenine, kynurenic
acid, xanthurenic acid and 3-hydroxyanthranilic acid were first detected and traced
under ultra-violet light (3655 A). The chromatograms were then sprayed with
Ehrlich's aldehyde reagent to develop the coloured spots for 5-hydroxyindole
acetic acid, kynurenine and 3-hydroxyanthranilic acid.
Semiquantitative evaluation of the chromatographic spots

This was conducted by comparing the areas of the chromatographic spots for
the metabolites obtained from the urines with those obtaned from the known
standards. Area measurements were carefully conducted with a planimeter and
the relative concentrations were derived from Fisher's equation (Fisher, Parson
and Morrison (1948) as follows:

A = b. log c + d

Where A = area of the spot; c = the concentration of the material on the spot;
b and d are constants which are dependent on the length of the run and condition of
colour development as well as on the particular characteristics of the pore pattern
of the filter paper sheet used. Since the standard and the unknown were run
under exactly the same conditions, the constants b and d can be neglected; and
thus if:

A1 was the area of a spot from a charcoal urine extract,

C1 was the concentration of the metabolite content of the spot

A2 was the area of the corresponding spot in the standard mixture and C2 was
the concentration of the metabolite standard content of the spot, then:

A1 log CQ
A2   log C2

A1 X log C2
..1og01-A2

The determination, however, was approximate and several factors were
involved including the length of run, irregularity in paper structure and area of
the applied substance to the paper.

RESULTS AND DISCUSSION

The results of the colorimetric, fluorimetric and chromatographic study of the
investigated metabolites are presented in the tables as follows:

The chromatographic separation of these metabolites gave sharp well identified
spots for kynurenine, anthranilic acid, 3-hydroxyanthranilic acid and 5-hydroxy-

594

URINARY EXCRETION OF TRYPTOPHAN METABOLITES

(1) bo       .     .   .

-'$ I         c       1-

c 00

" tD  o 00

.  4 .

4 )  C-   0 C o

Cd .-  0 ) 0 ) 0 1

=       o

* *

.:i   I e- Y e

a.  0H

_ Cs

Cs

.0
L   -

0          0

:e J  z co

CO CO
C r1  o  s0 C

L     0 .- ?4d

I  4 ) t   0   0   0

X N- 00

co CO 0- O0 CO   0

0  .  .  .1  .

o       N  N N
0 * N     .

C-0  00  a) 00 t-L

0  0  *) -  -  - O cO  0
o00 C- 001 01 001HH

O Co 010 01 Co CFo

I . . . . . . . I

1 00 t  C. s  ) Co 0
Co Co -I 00 Co 0 NN

_I 01 0 1 Co 0  _0010

O0 X C_1 N0 _ Co C.-

N IQ I I I- I z oo 0

.  Co  .  . 1 .   .   C..  0)

0t 001 t1 0 O 00 0 _0)

* Co N 01 0) O s C o

0  . . . . . . .

Co 00 0 01 eC0 0 01b

+ *    * 0 _CNo
*  0O  ~ O *  . .

0O -
Co  C o 0  Co 0)N

Co 010 , O0 0g 01

O0. )    N 00 00)

-o   *6655I*5t

00  0 0

0) 00 ()

O V _
0 '4 Co1
O    _I .

O O 0

00 00
0K1 Co 0)0 e

Co 00t

N _I I Iq

N1 Co C- C.

0 _t
00- 00 Co

01>N

01*0.1

0 O CO 00

- C.. 01

-1  00I

00 _I Co
H N1

00O

Co t. 00N

0000.

00 01O
Co 0) N1

oio 6 Co

00     0

to LO t0  0    N Lo     00 t- 0 ,)

LO b- & C; & N t: C& 4  4 4 4 =I

0)0 LO 00C0 l

'o o' C N 4
*  *  e o* N  ' .

t- 1 C      e- 00 Co

0 o 0 0 .. C...... 0*

.  . .C ..  .0.0.

*0    C o 0O 0   LO
o    * *  * O

o CO Co C- Co 01 o CoN

-       .  .o  .  Co.

?i ,  ?R c? ?   ? o - a - " - 0

x 0W(D  O       0

I I  l  Ml

Co Co _z 0

C. 000

C; CX  4   C0

"   -  Co Co
0-"00

01 0101

Co  0   o C

0) Co o    C0

4 _-4 CN
km km01l

Cs )    .       .

a)  0)~ ~ ~ ~ ~ ~ ~~~C

Cs 4  4).       C

'IO  CsCs0 c

0  7              0s  8

a-   a- m4 .   0 v  v v v   4v )

595

1.
01

04

.0
a)

o~ 9

eQZ

. tQ
* <Q
*4 S

e0

o

E-4

25

A. S. KHALAFALLAH AN) M. A. M. ABUL-FADL

-  *)  rf 4) -t   0 000  00  c   0o C 0 o

44  ?   0 0 0 0 0  t  '- 0 0 0 0 0   Ce   0   0   U0

C be

L           e q-

co       ae

i~~~~~~m C,

0  00  0o 0000o   0 0 o

.C) @   4t'.0   0 0 0  00 0 0 0  o  0   C00 0 0

0Q      000        0 OOeqO

P A   4 ) 0 w   e q o q   *  o o eqII I   -

to             - t 000

I 0    000000 CD   0  00  4  00  00  0
I  '000t-00 H 00t'.000  00000

to 00   t 00
0  -  -   ,-4

CS    .   .   .

<  1   0 0 0 0 0C

04

P44

000

co)o
I ooo

4 eq

eq  -   0

00
00 0 0

C  I  P )  00 *~ 0

oo CO

P' oH

P4).

Ec    =c4  1  II

L      00

- eq 00

km   t- -r   C  eq  00  -

eq o 0 o 0 0 0   00  0 0  00 0  0

e eq e eq - 4  - -

eq00        0  00 0 0 O

. 0   eq    .~ e   .  eq  0

r- COC bC    bC

Lo  co  000  0  CO 00 LO

eq

*  00  .  *o  t o  . 0 .00

eq.

N0  C- .  eq4 -4   CO  0 0 C

o         e qo4  eq

cq  00I -    e    ? 0

000 _I 1        N

cl eo   oh a o ol

*  00  0   eq   -  eq  e  00

r-o   -  r-A   rA   eq  e

-4 r-    r- O I  rI  r O
0     0  00  -  0   "

*  .  . * 0 .   * . .

0  0_4    0   O

O  00  C      00

e q>   .  . 0 .   0 0 0

eqt : 00 00 * 00 t.

t.   0 0  . 0  .   .   .  e q  .0 0

00 t 0000 - eq t. 00 0-

co t 00e O

-   *  *  0 0 0  eq  0   .   .

O      00  t -  00  0 0 0

.0  .  0 ' .   -3

6 4 -.   0 )   0   0   0 0   0 0 M0   0 0   0 0   0 0   0 0   0 0  0 0   0 0   0 0   0 0

o;  Q        oo   ea:e  o  o  :  .>c

o        4)

0      0

4 ~.     4)         0

4.A ) -?.*-

. .44 A   4 ' 4  ? o   t  S  5  > 0   2

C s IV  N Cs N  o  toos4)  -

1..                    0 00e

0  0X  0  0  0  0      0 V  V  V VCs Ca

Z*~~~~~~3~~~   ~ ~ ~ Cs      44r-

596

C3

0

0

C.)
x

4)

bs

4; a

~? 1

0
0

C$
4

A

* to

o ;
Co    40

m-%

. .o

*  42* ;

Z    Oz

$

.%D
. ez

o40 co 00tf
00 q 0o
4 :   Ct 1  00

000?
oo o 00 l
or o:

cq V 000
e4 q 00 o

0000o
s- o eq -

eq

IoOII I

* .eq.

b t D LO

o - U eq
q eq e

N   00N

. 00-

00 - 0

orHO eq
- of 00
*      0

I  _I  _I

~. .~ . -

* . . eq

Ci 00 0

URINARY EXCRETION OF TRYPTOPHAN METABOLITES

E t  .0 o~                          I

o 9 - x  0=  0  "t  C   0  x   =   0  0   N  0   0   m   10  0

1  0 10-  .  .  .. *.

H   m                 - O

ce _- --   -

0  0

E--

._ ~ ~   0 O  XO  N  N m  CX  N m  - 1 t

.       . . . . . . ...  .  .  .  .

*-

%OO 50Oe---0--O-------O_

O ~ ~ ~ ~ ~ ~~~~~c cq aqa Ocl l

;", ~. . . . . . . . . . . . . . ..
: ~ ~ ~ ~ (  C)  C) 00 .-o  00 to w o  Xo X

75 CD 00   00 C.  00 CD  -   t-   t- -  m >

c~ ~ ~~~~~c

0

o 0o

4-

C)     . .
4a  1

T$~~~~~~~~~~~~~
0

e ~~  ~  .   .   ..  .   .   .   .:  .   .   .   .   .   .   .   .

00       O   C

H   Q    ~. .   .   .   .   .   .   .   .   .

M     4 ."-a

>   0

t 0     33333

0 ~ ~ ~ ~ ~ ~

597

0 Q
4t

Co

. -

V O

VV

EsMQ.

598             A. S. KHALAFALLAH AND M. A. M. ABUL-FADL

TABLE IV.-Showing the Average Urinary Excretion of Tryptophan Metabolites (chromatographic

determination) in Normal and in Pathological Cases Before and After the L-Tryptophan Loading
Test

Tryptophan metabolite excretion (mg./21 hr.)

_~~~~~~~~ -                                         5

Diagnosis
Normals

Simple bilharziasis

Bilharzial hepato- .

splenomegaly

Bilharzial bladder

Cancer

Cancer cervix
Cancer breast

Cancer prostate
Cancer lip

Cancer larynx
Cancer thyroid
Cancer tongue

Malignant tumours

of the brain
Cancer bone
Cancer liver

Cancer bronchus
Cancer rectum

No.

of cases

8
8
8

5-Hydroxy-

indole

acetic acid

Before After

8-8
10-8  21-9
12-3  25-0

3-Hydroxy-
anthranilic

acid

Before After

2-0
1-9   3-6
1-7  3-3

Kynurenine
Before After

4-6   13-5
4-1   10-0
3-4    7-6

Kynurenic

acid

Before After

5 9   38-6
7-6  65-0
8-1  61-1

Xanthurenic

acid

Before After

2-4   10 5
2-2   10-9
3-0   12-9

8      18-2   30-6    4-6  22-1      3-8   11-1     4-3   38-0     1-6   7-5

3
3
3
3
3
3
3
3

3
3
3
3

14-7
38-7
57 -7

28-9

8-7

28-0
81 -0
105-3

9-3

56-9

1-5
1-9

15-7   30 3
14-5   28-6
15-8   30- 3

3-4
0-7
2-

1-5
3-6
. 2

21

4-2
4-0
5-5
5 0
3-6
3.4
4 0
3-3

13-9
11 -6
15-7
15- 1
10-9
9.9
14-9
11-1

7 9
6-1
7 9
7-2
5-9
8-6
7-5
7-6

56-3
62-8
68-0
60-0
45- 0
54-3
64-3
90-8

2-4
1 -8
1 -4
1-9
2-1
2-4
2-5
2-6

4-8   13-4    8-0   74 0     2-3  11-1
3-3    9-6    6-5   48-3     1-2   6-1
6-4   20-2    5-5   48-6     2-3  10-5
2-9   10-2    5-3   42-6     1-5   7-2

indole acetic acid. Kynurenic and xanthurenic acids on the other hand, the RB

values of which are so close to each other (0-55 and 0-56 respectively in butanol-
acetic acid water (Partridge, 1948)), were almost overlapping, and thus their proper
semiquantitative estimation was not satisfactorily achieved. Nevertheless, an
approximate idea of the quantities excreted of these two metabolites in the urine
was always reasonably obtained by a more careful chromatographic study.
Indole-3-acetic acid, however, was not chromatographically detected in any urine
whether normal or pathological, before or after administration of tryptophan.
This metabolite seems to have a high affinity for the deactivated charcoal on which
it was easily adsorbed but not possibly eluted by the phenol, and its quantitation
was thus confined to the colorimetric methods. Besides the latter metabolite,
the detection of 5-hydroxyindole acetic acid, anthranilic acid and 3-hydroxy
anthranilic acid in the urines collected from normal as well as from certain patho-
logical cases, was not amenable to the above chromatographic technique.

It seems that these metabolites were excreted in such low concentrations as to
escape detection in the eluate. Their quantitation was also confined to the colori-
metric methods.

The results obtained for each of the above mentioned metabolites are discussed
below.

Anthranilic acid

The normal excretion of anthranilic acid as detected colorimetrically was
found to range from 0-14 to 0-9 with an average of 0-5 mg. /24 hour urine.

In bilharziasis, whether simple or complicated, no apparent variation from
normal was detected. In bladder cancer, the average excretion of this metabolite

8-4
7-6
6-0
8-5
8-6
9-3
10-7
10-3

URINARY EXCRETION OF TRYPTOPHAN METABOLITES

was found to be increased to about six times the average normal value. No rise
to such an extent was observed in any of the malignancies studied which involve
organs other than bladder. For example, in cancer of the cervix, the thyroid, the
breast and in malignant tumours of the brain, there was mild elevation in the urinary
excretion of anthranilic acid but the highest level was only a little above the
maximum normal excretion.

After the oral administration of tryptophan the average rate of excretion of
anthranilic acid in the urine as compared with the same before loading test, i.e.
the A /B* ratio, was 1 8 in normals, 1-8 to 1 9 in bilharzial infection, while in bladder
cancer, this ratio was 2 7, although it did not exceed 1.8 in any of the other forms
of cancer examined. This shows clearly that although the anthranilic acid is only
excreted in small quantities in urines of normals and malignancies, yet its relative
increase is, so far, a characteristic feature of bladder cancer.

Such results for bilharzial bladder cancer seem to be in agreement with those
described by Boyland and Watson (1956) and Price (1958), working on bladder
cancer in England and America respectively where this disease is apparently of
industrial origin. Thus the metabolic turnover in this disease, as showil by
the anthranilic acid excretion, seems to be the same whether the suspected cause
is industrial or bilharzial. The only difference is that the figures mentioned by
the British and American authors are slightly higher than those found in this
country. This also applies to the level in normal subjects. Thus the relatively
low anthranilic acid urinary excretion in this country might possibly be attributed
to differences in climatic, racial or nutritional habits.

3-Hydroxyanthranilic acid

The normal urinary excretion of 3-hydroxyanthranilic acid was found to range
from 045 to 24 mg. with an average of 1-4 mg./24 hour urine. In bilharziasis,
whether simple or complicated, excretion of this metabolite was increased to about
twice the normal average.

In bladder cancer, however, the average urinary excretion of this metabolite
was found to be increased to about 8 times the average normal values.

In cancers of the breast, malignant tumours of the brain and reticulum cell
sarcoma of the liver, the urinary excretion of 3-hydroxyanthranilic acid ranged
from 2-7 to 3-2 mg./24 hour and the average excretion level was only twice as
much as the normal.

In malignancies involving the prostate, the lip and the larynx the average
urinary excretion of this metabolite was greatly decreased below the normal
average level, particularly in prostatic cancer where urinary 3-hydroxyanthranilic
acid was almost absent in some cases.

Other types of cancer did not seem to produce any significant variation from
the normal level of excretion.

Following the oral administration of tryptophan the average rate of excretion
of 3-hydroxvanthranilic acid, as compared with same before the loading test
(i.e. A/B ratio), was 1-7 in normals, 2 in bilharzial infection, and 5-2 in bladder
cancer.

In malignancies involving the cervix, breast, lip anid thyroid this ratio ranged

* A 'B ratio = After/Before test.

599

A. S. KHALAFALLAH AND M. A. M. ABUL-FADL

from 2.0 to 2*2, while in the remaining types of malignancies the ratio was within
normal limits.

This shows clearly that the maximum excretion of 3-hydroxyanthranilic acid
is characteristically obtained in bilharzial bladder cancer, although the amount
usually excreted is again less than that recorded abroad, possibly for the same
reasons mentioned under anthranilic acid.

These results are in fair agreement with those reported by Boyland (1956)
on the aniline bladder cancer and bladder cancer in the general population of
England.

Hence, the tryptophan metabolic disorder of this disease, as far as 3-hydroxy-
anthranilic acid excretion is concerned seems to be the same whether the cause is
industrial or bilharzial.

Jndole-3-acetic acid

The normal urinary excretion of indole-3-acetic acid as determined colori-
metrically ranged from 3 13 to 16.5 mg. with an average of 7 0 mg. /24 hour urine.
The present chromatographic technique however failed to detect this metabolite
either in normals or in any of the pathological urines investigated.

No significant variation in the urinary excretion of this metabolite was noticed
in bilharziasis, whether simple or complicated, or in bilharzial bladder cancer.

In cancer of the prostate and of the larynx the average urinary excretion of
indole-3-acetic acid was markedly below the normal average. The other types of
cancers examined, on the other hand, did not show any significant variation from
the normal level of excretion of this metabolite.

After the tryptophan loading test the indole-3-acetic acid excretion varied in
normal subjects from 835 to 17-95 mg. with an average of 12.5 mg./24 hour (A/B
ratio = 1-9).

In bilharziasis, whether simple or complicated, and in bilharzial bladder cancer,
the picture remained still within the normal range. On the other hand, patients
with malignancies involving the breast, the tongue, the lip, the cervix, the rectum
and the thyroid gland seem to excrete more indole-3-acetic acid than did the nor-
mal subjects after the oral tryptophan test (A/B ratio ranged from 1.8 to 2.2).

In cases of cancer of the prostate and cancer of the larynx the excretion of
indole-3-acetic acid was still below that of the normal subjects similarly treated.
The remaining types of cancer showed no variation from normals after tryptophan
administration. Thus the tryptophan loading test confirmed the characteristic
property of prostate and larynx cancer as regards the diminished excretion of
indole-3-acetic acid in urine. It also revealed the tendency of certain other
malignancies towards increased excretion of this metabolite in the urine.

5-hydroxyindole acetic acid

The normal urinary excretion of 5-hydroxyindole acetic acid varied from 3-2
to 13-3 mg. with an average of 8-6 mg. /24 hour urine. In simple and complicated
bilharziasis the average urinary excretion of this metabolite ranged from 21-7 to
22 6 mg. /24 hour urine, showing a level about 3 times as high as that of the normal.

In bilharzial bladder cancer on the other hand, the average excretion of this
metabolite was increased to about 4 times the normal average.

As for the other malignant diseases, urinary 5-hydroxyindole acetic acid was

600

URINARY EXCRETION OF TRYPTOPHAN METABOLITES

considerably increased in larynx cancer, lip cancer and malignant tumours of
the brain to an extent exceeding that found in bladder cancer. The average
excretion of this metabolite in the above three conditions with respect to the
normal average excretion amounted to about 10, 7 and 5 fold increases respec-
tively. The excretion of this metabolite in cancer of bone, liver and bronchus
was of about the same order as that in the case of bilharziasis. In cancer of the
rectum, tongue and breast, on the other hand, the level of excretion of 5-hydroxy-
indole-acetic acid was found to be persistently on the lowest side of the normal
range.

Boyland, Gasson and Williams (1957) have also reported on the increase in the
excretion of this metabolite in cancer of the larynx and bronchus. It is of interest
to mention here that, during our studies on pellagra in Egypt, we have noticed a
marked increase in the urinary excretion of this metabolite in untreated pellagra
cases to an extent comparable to that found in bilharziasis, which diminished after
treatment with adequate doses of nicotinamide (work in progress, unpublished
yet).

The tryptophan loading test showed also a pronounced increase in the excretion
of this metabolite in the urine of the above mentioned patients with an already
elevated excretion before the test, but the A /B ratio was only a little above normal
except for the cancer of the larynx cases where the ratio was more than 4 times
that of the normal.

The increase in the excretion of 5-hydroxyindole-acetic acid has been said to
be pathologically related to the tumour origin since the bladders of bilharziasis
usually show squamous metaplasia and the cancer is usually a squamous cell
carcinoma. The larynx, lip, liver and bronchus all develop squamous cell
carcinoma and produce marked increase in the urinary excretion of 5-hydroxy-
indole acetic acid.

Cancer of the cervix, however, though frequently of squamous cell type, yet
did not show any increase, whereas the brain which is ectodermal in origin and
with a different histological type of tumour from the squamous cell carcinoma
showed marked increase in the urinary excretion of this metabolite.

This shows clearly that the type of the malignant cell does not seem to be a
primary factor affecting the urinary increase in the 5-hydroxyindole-acetic acid
excretion, and that certain other factors which possibly involve the cell nutrition
and metabolism must be considered.

Kynurenine

The normal urinary excretion of kynurenine was found to range from 4 to
9-2 mg. with an average of 5 9 mg./24 hour urine. Somewhat lower results were
obtained by the chromatographic technique (range 3 0 to 6; average 4.6 mg./24
hour urine). In simple bilharziasis and bilharzial hepatosplenomegaly no signi-
ficant deviation from the normal level was detected by either the colorimetric
or the chromatographic method. In bladder cancer, an increase in the excretion
of this metabolite to about twice the average normal level was found. Slight
elevation in the kynurenine excretion was noticed in cancer of the cervix, breast,
prostate and bronchus. In cancer of the rectum, however, the excretion level
was rather low, whilst in the other types of malignancy examined no significant
variation from normal was noticed.

601

A. S. KHALAFALLAH AND M. A. M. ABUL-FADL

Colorimetric and chromatographic methods of analysis of this metabolite
showed agreement, although the values obtained by the latter technique were
still lower than those obtained by the former. The values described for kynure-
nine excretion in industrial bladder cancer (Price, 1958) seem to be near to those
recorded in bilharzial bladder cancer in the present work.

After the tryptophan loading test, marked elevation in the urinary excretioii
of this metabolite was obtained in cancer of the bronchus, cancer of the prostate,
cancer of the lip and in bladder cancer in decreasing order though the highest
A /B ratio was obtained in the last. In the other types of cancer examined the
level of excretion was almost within normal limits.

Kynurenic acid and xanthurenic acid

These two metabolites were determined by fluorimetric and by chromato-
graphic analysis.

Fluorimetrically, the urinary excretion of kynurenic acid in normal Egyptians
was found to range from 6*5 to 15*9 mg. with an average of 11L7 mg./24 hour;
xanthurenic acid excretion on the other hand ranged from 4 to 13 mg. with an
average of 9.1 mg./24 hours.

Using the chromatographic technique, kynurenic acid was detected in the
urine of normal subjects in amounts ranging from 5.1 to 6-9 mg. with an average
of 5*9 mg./24 hours.

Xanthurenic acid was excreted from 1 6 to 2*8 mg. with an average level of
2*4 mg./24 hours.

The marked discrepancy in the results between the fluorimetric and the
chromatographic analysis is due, as previously mentioned, to the close R, values
of these two metabolites, besides the possible loss involved in the latter technique.

In simple bilharzial infection, a slight elevation in the level of both the meta-
bolites was effected. The elevation becomes more evident in bilharzial hepato-
splenomegaly, in which the average excretion level reached one and half times the
normal average excretion for each of the two metabolites.

In bladder cancer, on the other hand, the average excretion is surprisingly
reduced below the normal average level of excretion of both metabolites.

Carcinomas of larynx and breast showed also relatively lower levels of excre-
tion, while other types of malignancy did not show significant variation from the
average normal values.

The tryptophan loading test in normal subjects resulted in an excretion of
65 to 85 mg. of kynurenic acid with an average of 74.3 mg./24 hours, while
xanthurenic acid excretion ranged from 34 to 50 mg. with an average of 45-6
mg. /24 hours as determined fluorimetrically. In bilharzial patients, whether
suffering from simple bilharzial infection or complicated with hepatosplenomegaly,
there was an increase in the urinary excretion of these two tryptophan metabolites
amounting to about 1.5 times the normal.

Cancer of the bladder on the other hand showed a relatively lower excretion
level than normal for kynurenic acid only, but not for xanthurenic acid. The
excretion of the latter after tryptophan in bladder cancer was within the normal
level. Cancer of the larynx produced the lowest level of excretion for both kynu-
renic and xanthurenic acids. Other types of malignancy behaved within the
normal range.

602

URINARY EXCRETION OF TRYPTOPHAN METABOLITES     603

SUMMIARY AND CONCLUSION

1. Colorimetric, fluorimetric and chromatographic determinations of anthra-
nilic acid, 3-hydroxyanthranilic acid, indole-3-acetic acid, 5-hydroxyindole acetic
acid, kynurenine, kynurenic acid and xanthurenic acid have been carried out in
normals, patients with bilharziasis (simple and complicated), bladder cancer, and
malignancy involving certain organs other than the bladder.

2. The change which is brought about as a result of bilharzial infection, whether
simple or complicated, seems to involve an increase in 5-hydroxyindole acetic
acid (about 3 times normal), 3-hydroxyanthranilic acid (about doubled), kynurenic
acid and xanthurenic acid (about 1.5 times). No apparent change was detected
in the excretion of indole-3-acetic acid, anthranilic acid and kynurenine.

3. In bilharzial bladder cancer the characteristic pattern of excretion of these
metabolites can be summarized as follows:

(a) A marked increase in: 3-hydroxyanthranilic acid (about 8 times),
anthranilic acid (about 6 times); 5-hydroxyindole acetic acid (about 4
times); and kynurenine (about 2 times).

(b) Kynurenic and xanthurenic acids showed characteristic diminution
below the normal average (about 50%0 reduction).

(e) Indole-3-acetic acid excretion showed no significant variation from
the average normal.

4. The study of the levels of excretion of these metabolites in malignant
disease involving bone, the brain, breast, bronchus, cervix, larynx, lip, liver,
prostate, rectum, thyroid gland and tongue showed a marked elevation in the
level of excretion of 5-hydroxyindole acetic acid in relation to the average normal
excretion per 24 hours (larynx cancer (10 times), lip cancer (8 times) and malignant
tumours of the brain (5 times)).

5. The characteristic rise in the urinary excretion of anthranilic acid and
3-hydroxyanthranilic acid, together with the diminution in the excretion of
kynurenic acid and xanthurenic acid which was reported in bladder cancer, was
not detected in any of the other forms of malignancy which were examined.

6. The tryptophan loading test was conducted on a number of cases repre-
senting each group of the above mentioned normal and pathological cases. The
test has proved its value in assessing the above mentioned findings. This was
particularly evident in bilharziasis, cancer of the bladder and cancer of the larynx.

7. The excretion pattern of the above mentioned tryptophan metabolites in
bilharzial bladder cancer in Egypt seems, so far, to be close to that described by
other workers for industrial bladder cancer abroad.

The authors wish to thank sincerely Professor A. Haddow and Professor E.
Boyland, of the Chester Beatty Cancer Research Institute, for a gift of L-trypto-
phan.

REFERENCES

ABUL-FADL, M. A. M. AND KHALAFALLAH, A. S. (1961) Brit. J. Cancer, 15, 479.

ALLEN, M. J., BOYLAND, E., DUKES, C. E., HORNING, E. S. AND WATSON, J. G. (1957)

Ibid., 11, 212.

BOYLAND, E., GASSON, J. E. AND WILLIAMS, D. C. (1957) Brit. J. Cancer, 11, 120.
Idem AND WATSON, G. (1956) Nature, Lond., 177, 837.

604           A. S. KHALAFALLAH AND 1I. A. M. ABUL-FADL

BOYLAND, E. AND WILLIAMS, D. C. (1956) Biochem. J., 64, 578.
DALGLIESH, C. E.-(1955) Ibid., 61, 334.

DUNNING, W. F., CURTIS, M. R. AND MAUN, M. E. (1950) Cancer Re8., 10, 454.
FISHER, R., PARSON, D. AND MORRISON, G.-(1948) Nature, Lond., 161, 764.

MACFARLANE, P. S., DALGLIESH, C. E., DUTTON, R. W., LENNOX, B. W., NYHUS, L. M.

AND SMITH, A. N.-(1956) Scot. med. J., 1, 148.
PARTRIDGE, S. M.-(1948) Biochem. J., 42, 238.

PRICE, J. M.-(1958) Univ. Mich., med. Bull., 24, 461.

SATOH, K. AND PRICE, J. M. (1958) J. biol. Chem., 230, 781.
TOMPSETT, S. L.-(1959) Clin. Chim. Acta, 4, 44.

WEISSBACH, H., KING, W., SJOERASMO AND UDEN FRTEND., S. (1959) J. biol. Chem.,

234, 81.

				


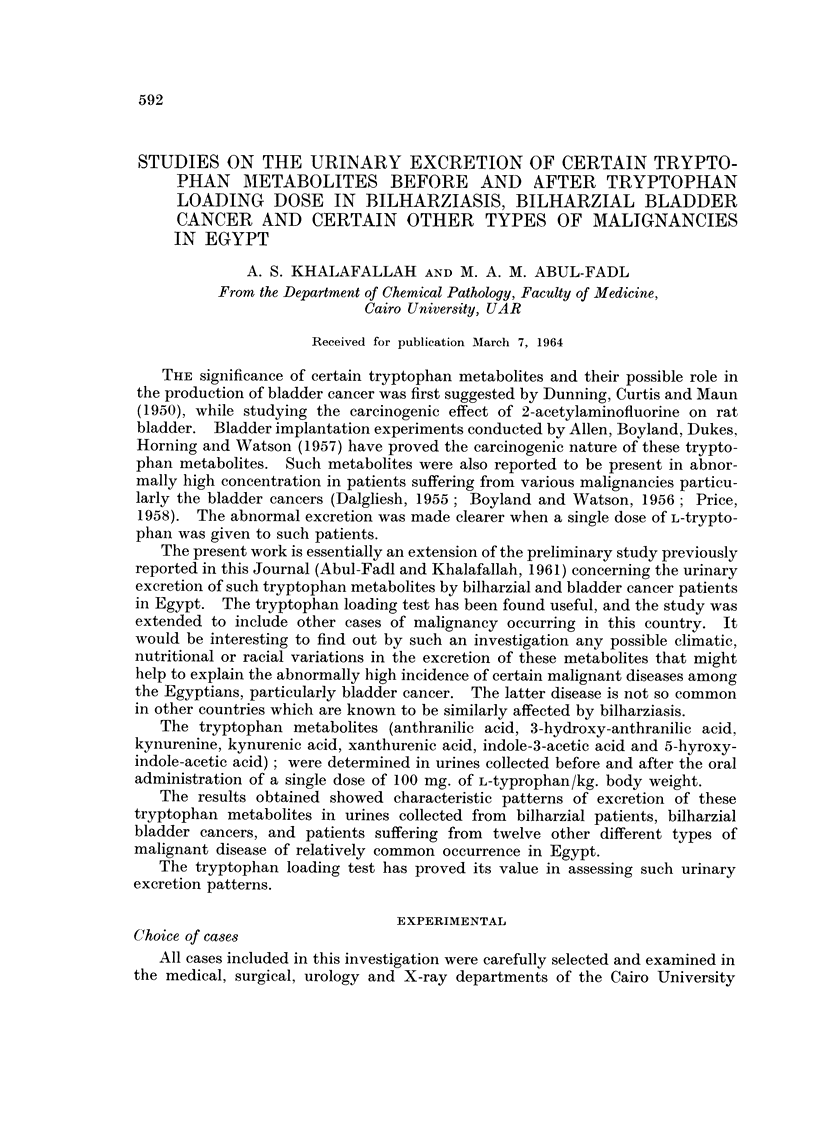

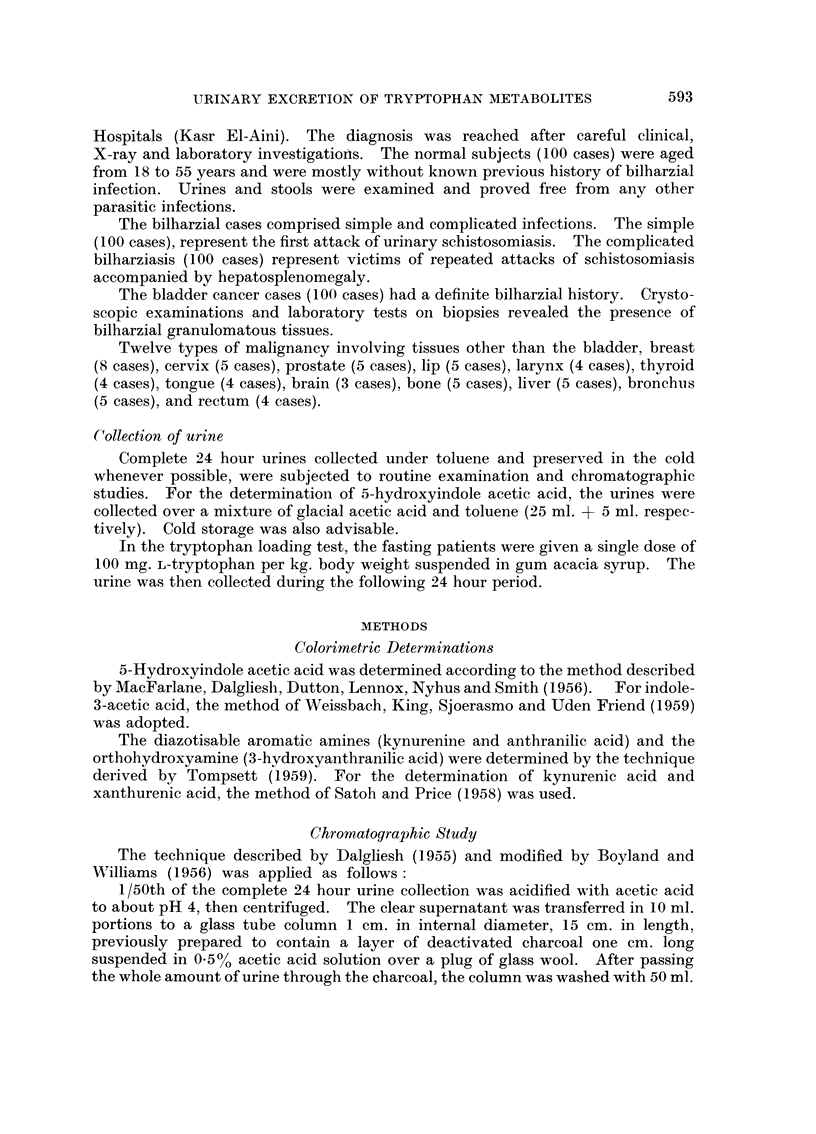

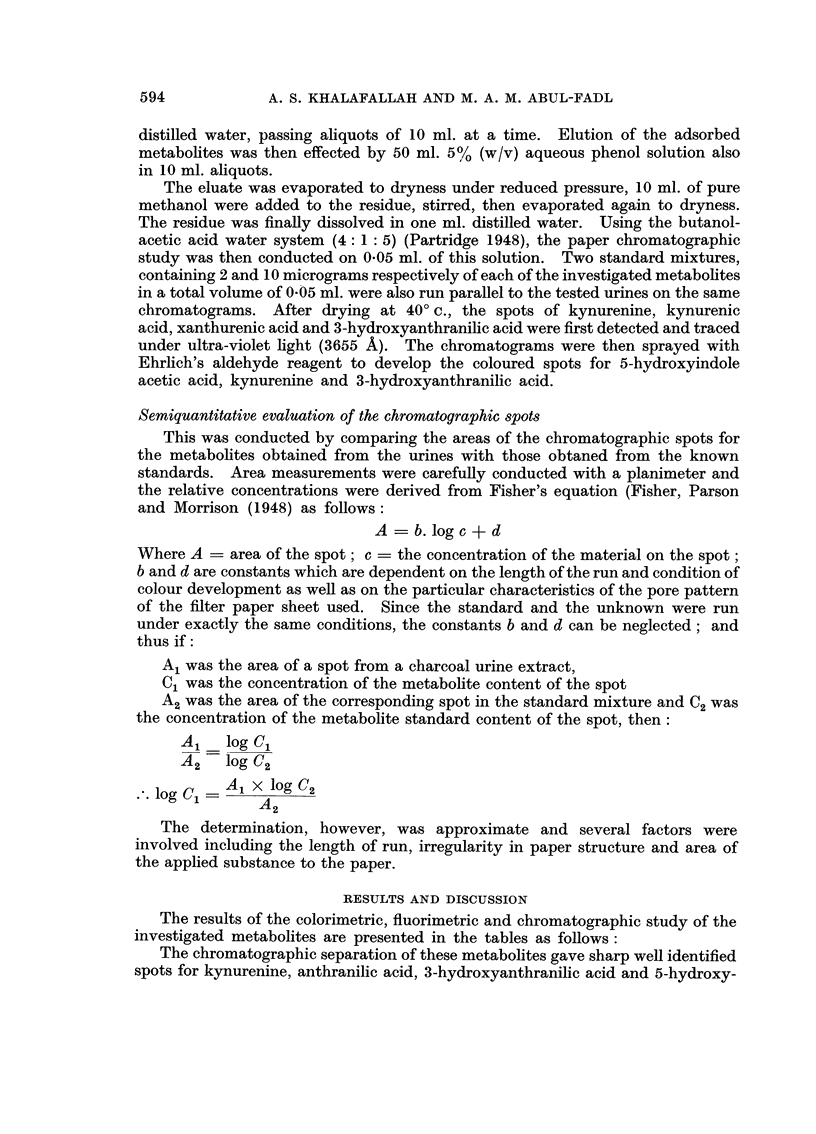

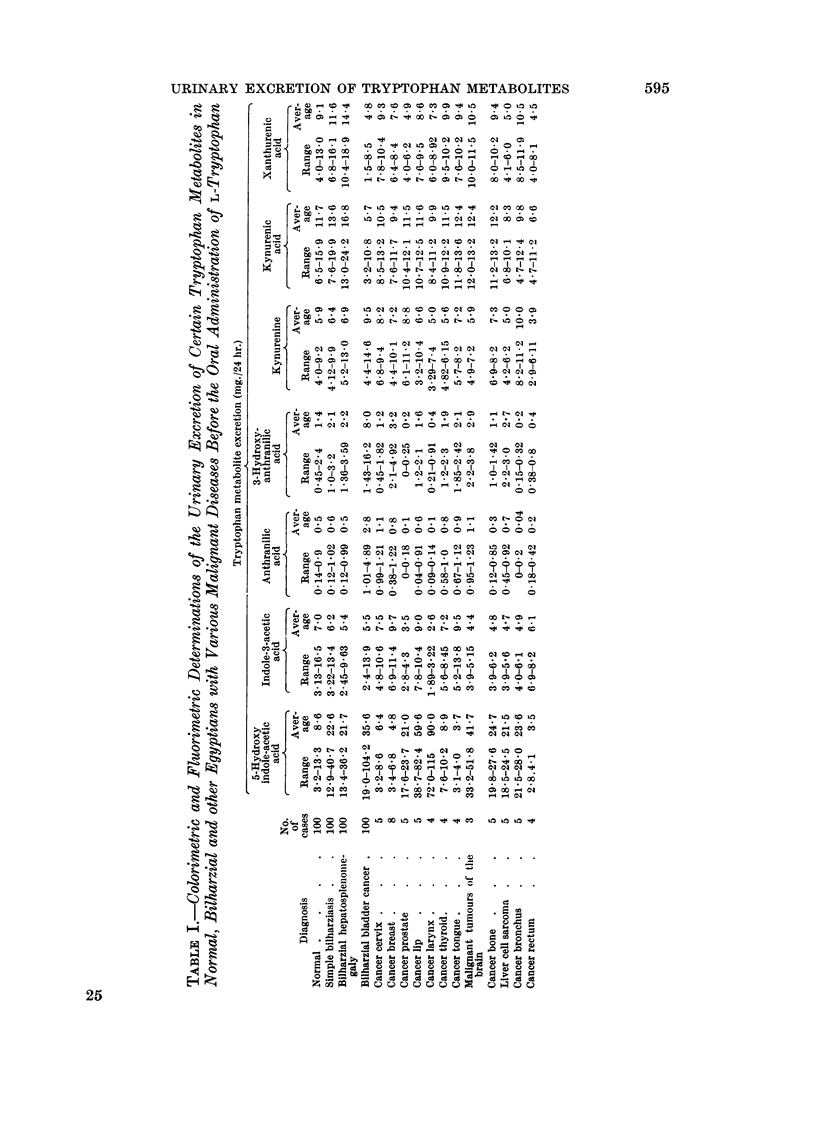

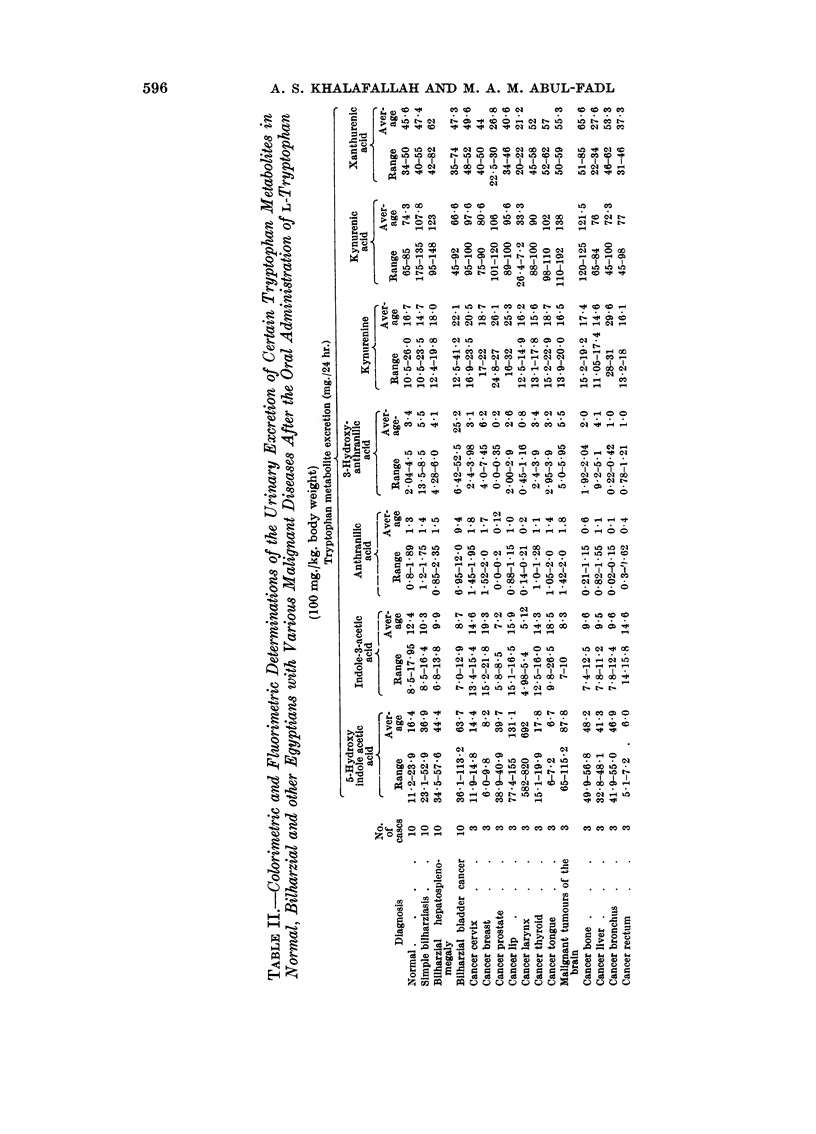

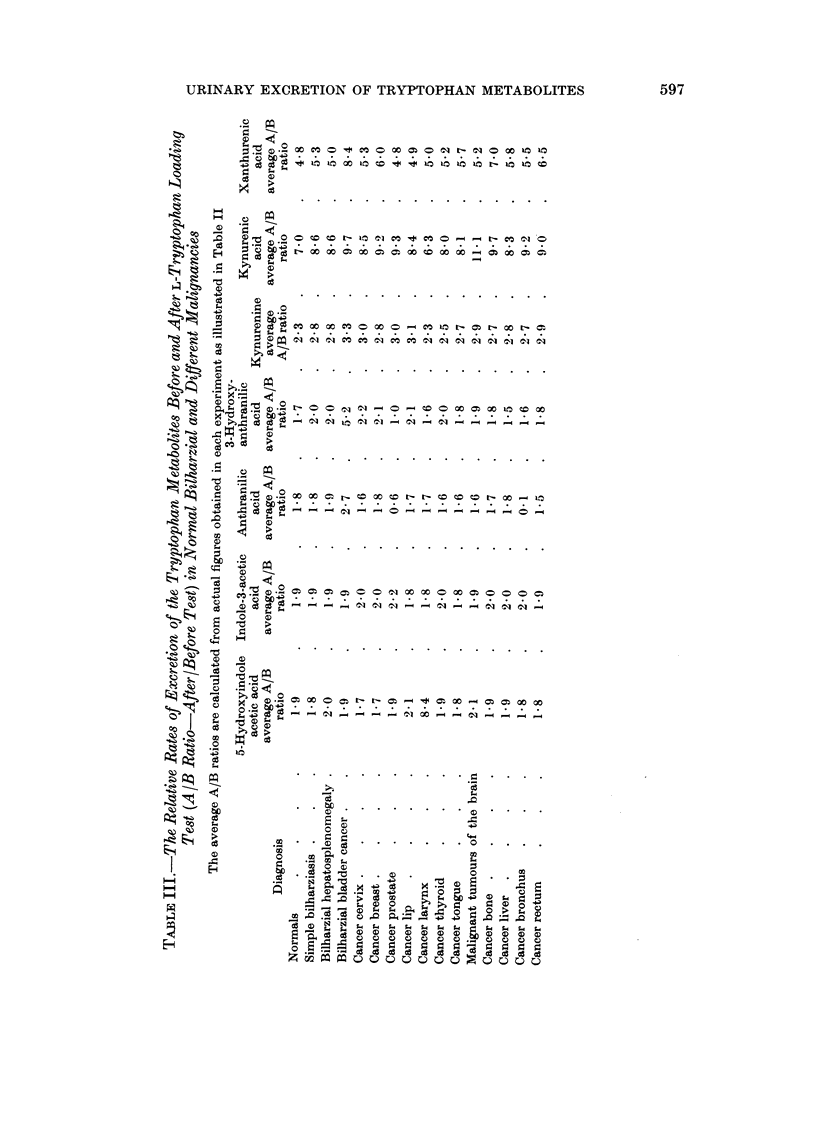

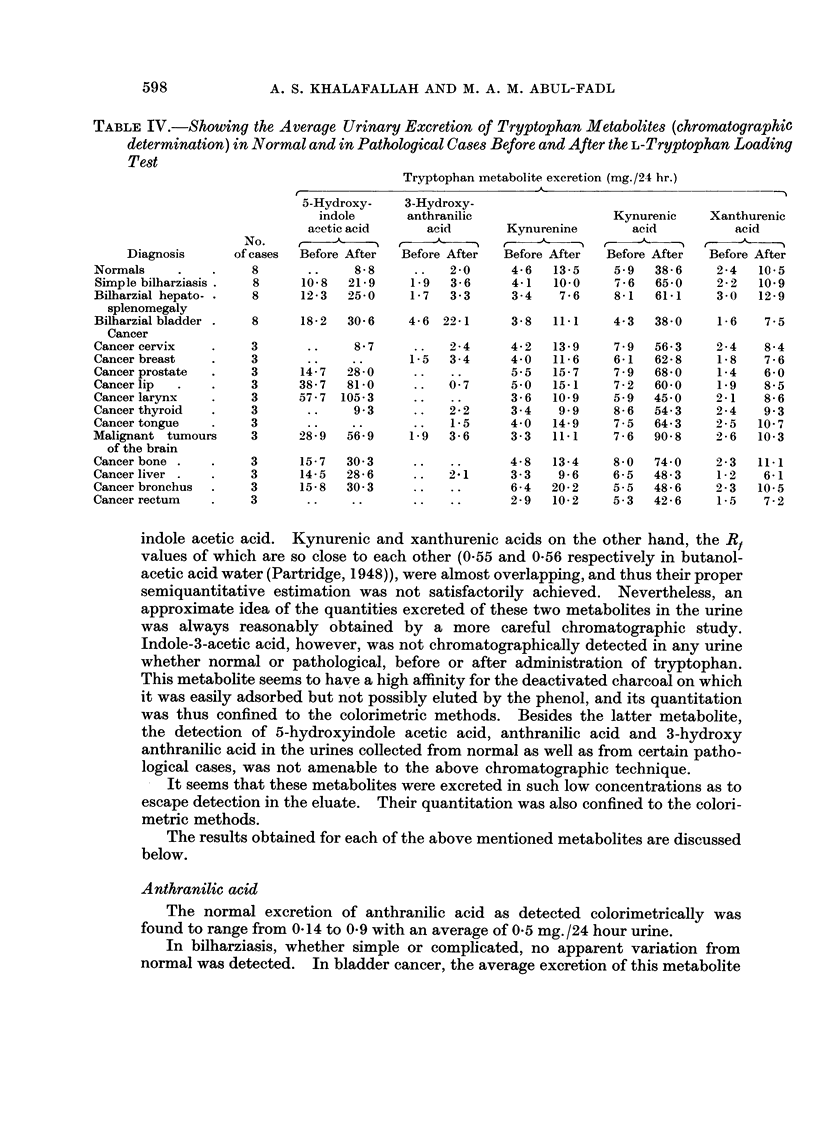

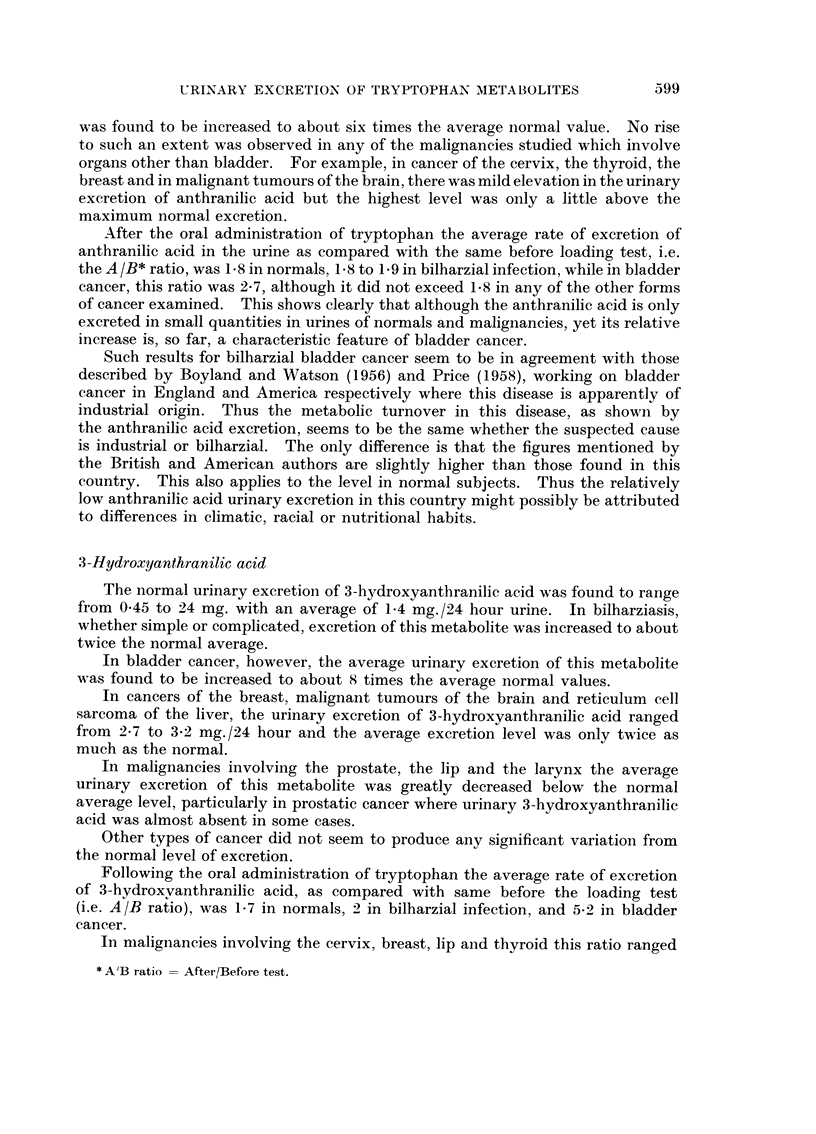

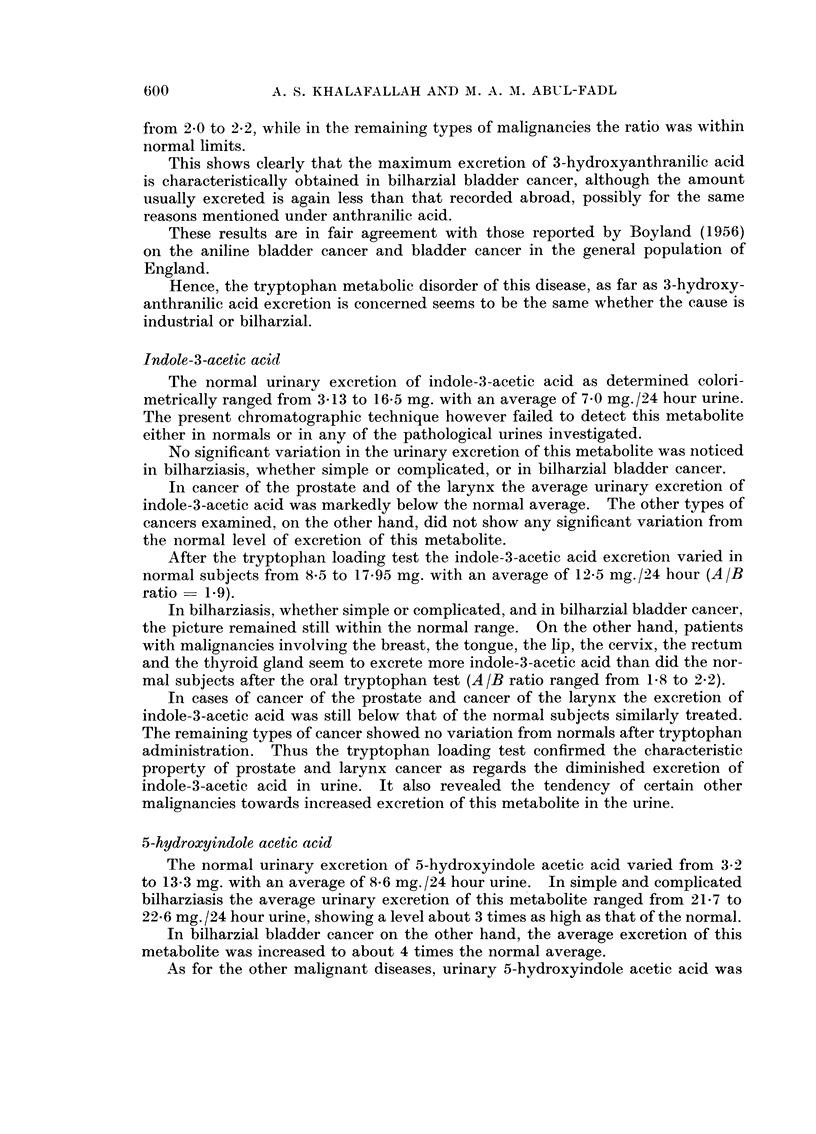

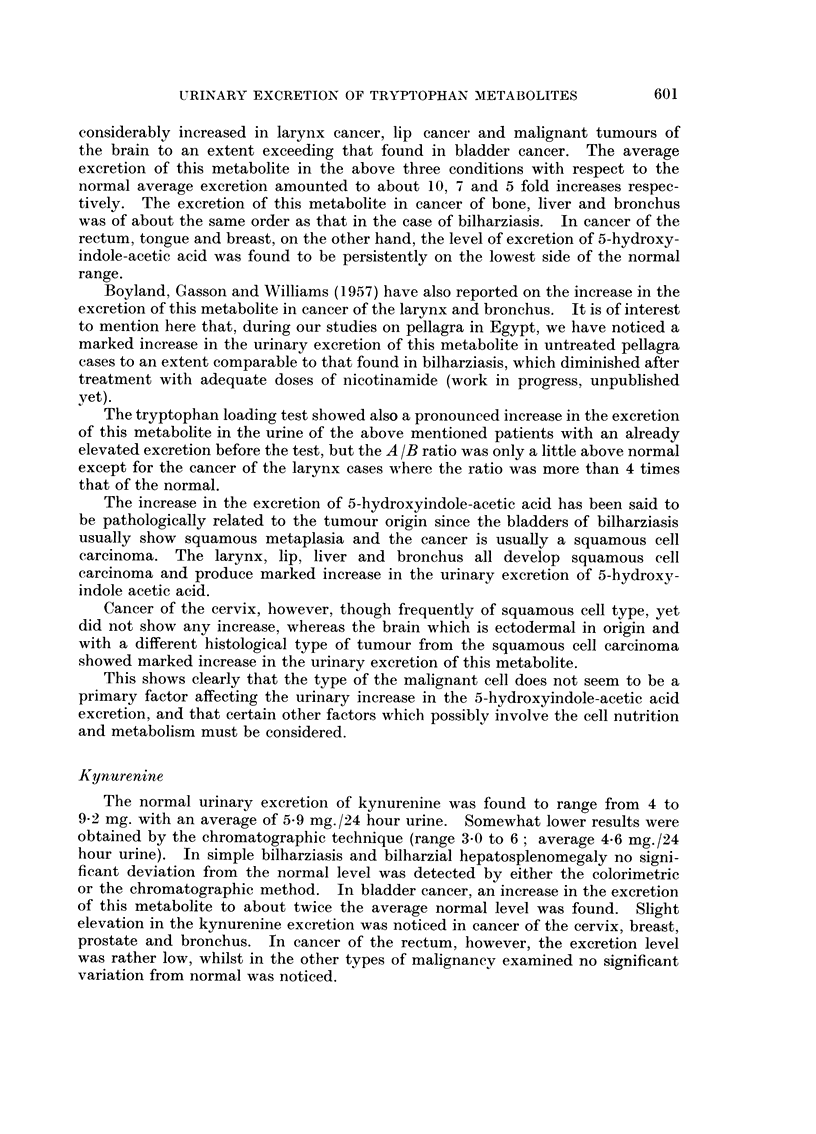

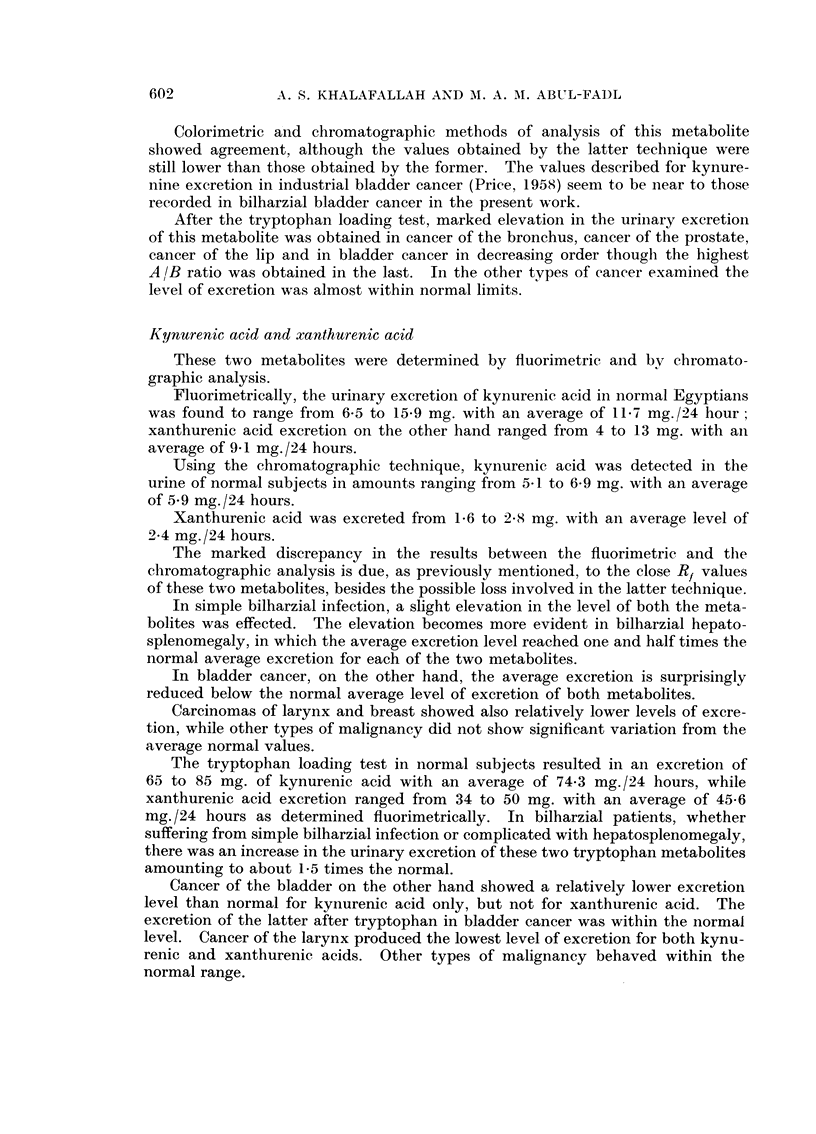

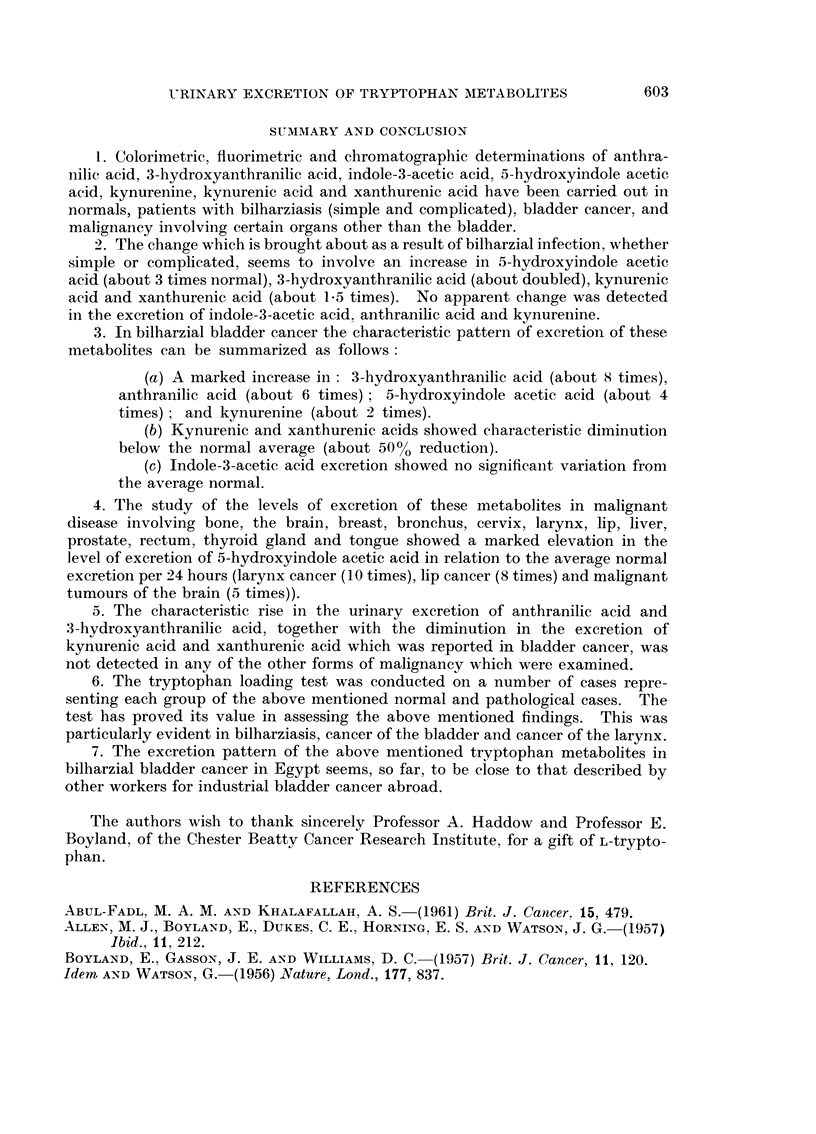

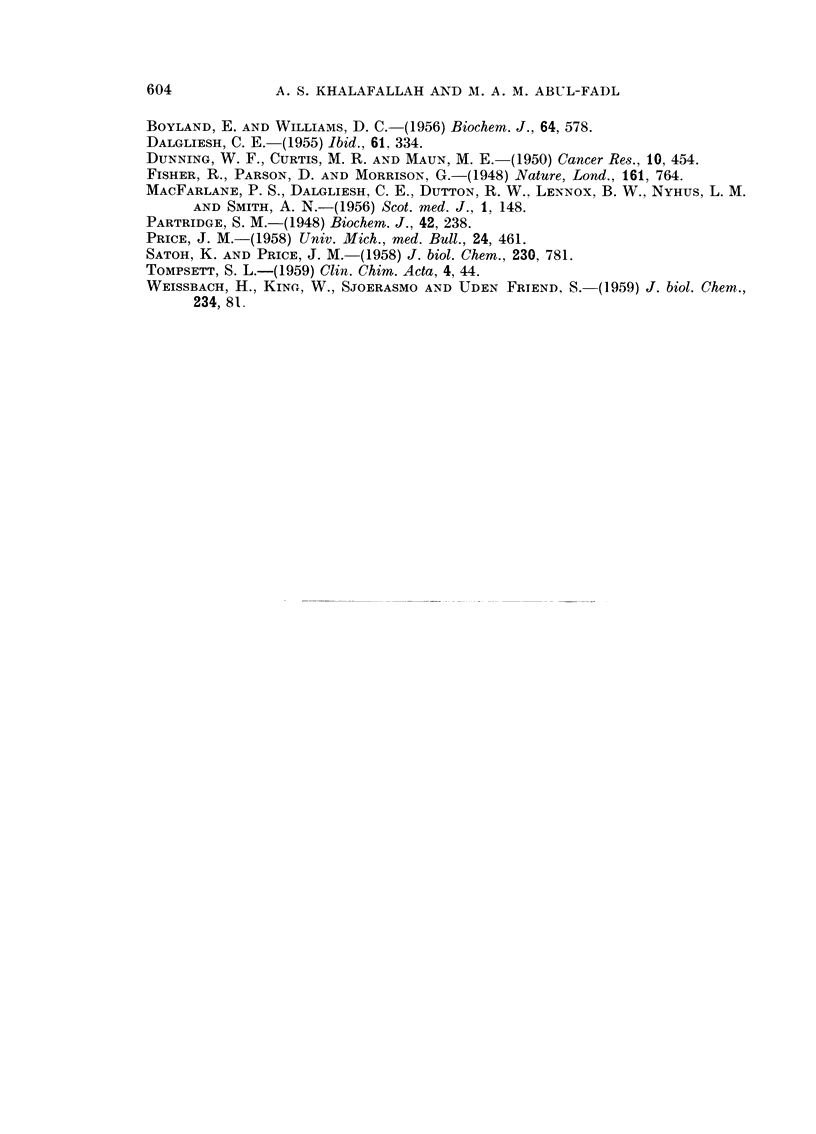

